# Exact Rovibronic Equivalence of the Adiabatic and Diabatic Representations of N‐Coupled State Diatomic Systems

**DOI:** 10.1002/jcc.70181

**Published:** 2025-07-22

**Authors:** Ryan P. Brady, S. N. Yurchenko

**Affiliations:** ^1^ Department of Physics and Astronomy University College London London UK

## Abstract

The Born–Oppenheimer approximation assumes nuclear motion evolves on single, uncoupled potential energy surfaces, widely used to solve the time‐independent Schrödinger equation for atomistic systems. However, for near‐degenerate same‐symmetry electronic states, avoided crossings in the potential energy curves occur and non‐adiabatic couplings (NACs) become significant. In such cases, the adiabatic approximation is unsuitable for high‐resolution spectroscopy. A unitary transformation to the diabatic representation can eliminate NACs, resulting in smooth molecular property curves that may cross. Computing this adiabatic‐to‐diabatic transformation (AtDT) is desirable but non‐analytic for multi‐state coupled systems, necessitating numerical solutions. It remains unclear if current methods yield numerically exact AtDTs ensuring rovibronic energy level equivalence between adiabatic and diabatic pictures. We demonstrate (for the first time) numerically exact equivalence of adiabatic and diabatic representations for N‐state diatomic molecules using ab initio data for $N_{2}$, CH, and a model 10‐state system. We show how the equivalence can be efficiently used to assess the importance of non‐adiabatic effects and the impact of omitting them when computing rovibronic energies of diatomic molecules. The adiabatic and diabatic representations of the spectroscopic model, including all coupling terms, have been implemented in the diatomic code duo.

## Graphical abstract

A novel method demonstrates the numerical equivalence of adiabatic and diabatic representations in N‐state coupled diatomic systems. This crucial finding provides an efficient and accurate way to assess the importance of non‐adiabatic effects, which are often disregarded without rigorous quantitative checks.

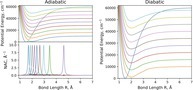

## 1 Introduction

**FIGURE 1:**
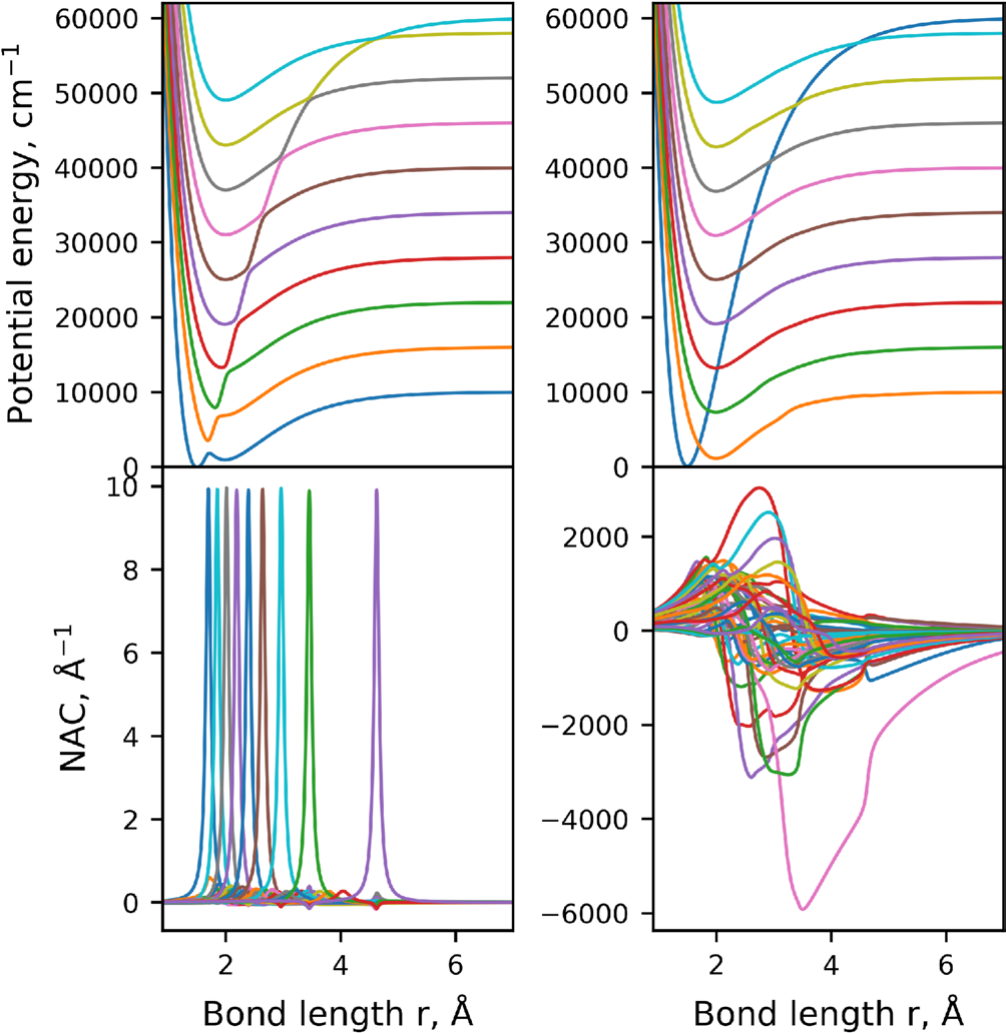
Illustration of the diabatisation of synthetic 10‐state coupled system: Adiabatic PECs (top left), NACs (bottom left), diabatic PECs (top right), and DCs (bottom right).

To address the longstanding challenges in accurately modeling nonadiabatic effects in diatomic molecular systems, we present the first demonstration of numerical equivalence between the adiabatic and diabatic representations for multistate coupled systems, providing insights critical for high‐resolution spectroscopy and beyond. Non‐adiabatic effects play a critical role in the photodynamics of many molecules and in physicochemical processes [1, 2, 3, 4, 5, 6, 7] which alter electronic structure and nuclear dynamics. These effects are significant in areas such as atmospheric chemistry and astronomy, where interactions involving free radicals and open‐shell molecules with spatially degenerate electronic states are common [8, 9, 10, 11, 12]. However, in the efforts to model near equilibrium properties of many molecules nonadiabatic effects are ignored and the Born‐Oppenheimer (BO) approximation, which assumes nuclear motion to be much slower than the corresponding electronic motion, has been extensively used and generally yields accurate results [6]. The BO and, via extension of the nuclear momentum operator by the diagonal Born‐Oppenheimer corrections (DBOCs), the adiabatic approximation means nuclear dynamics evolve on single uncoupled potential energy surfaces or potential energy curves (PECs) [8]. While this assumption works when states are energetically well separated, approach of electronic states of the same symmetry breaks this approximation, where PECs exhibit avoided crossings (see, e.g., Figure 1). It is in these cases where inclusion of the derivative couplings (DDRs), or nonadiabatic couplings (NACs), and hence relaxation of the BO approximation, is required to correctly describe the nuclear dynamics across the web of complex electronic structure. We note here that use of the term ‘adiabatic representation’ now means inclusion of all DDR couplings and avoided crossing potentials. The term DDR is the commonly used contraction of $\frac{d}{dr}$, where r is the diatomic nuclear coordinate. Herein, first and second DDR coupling refers to first‐ and second‐order radial derivative couplings, respectively.

Nuclear motion can be described using either the adiabatic or diabatic representation. The adiabatic representation employs Born–Oppenheimer potential energy curves (BO‐PECs), DBOCs, and off‐diagonal DDRs. The diabatic representation is then achieved through a unitary transformation of the adiabatic electronic wavefunctions, known as the adiabatic‐to‐diabatic transformation (AtDT) [13, 14, 15, 16, 17, 18, 19, 20, 21, 22, 23], which removes radial DDRs (coupling states of the same symmetry) at the cost of introducing diabatic couplings (DCs), allowing diabatic PECs to cross. In contrast to the adiabatic representation, the diabatic nuclear kinetic energy matrix is diagonalised (eliminating DDRs) and PECs are coupled by off‐diagonal DCs [21, 22, 23]. While the adiabatic representation diagonalizes the electronic Hamiltonian, the diabatic representation diagonalizes the nuclear kinetic energy.

The primary advantage of the diabatic representation lies in its smooth PECs and molecular property curves, such as the dipole moment [24]. This smoothness simplifies the generation of analytical models for constructing efficient contracted rovibronic basis sets. This is crucial for refining these curves to better match experimental data, as demonstrated by projects like ExoMol [25, 26, 27]. In contrast, the adiabatic representation's cusp‐like PECs and singular DDRs near degeneracies [8, 22, 23, 28] complicate integration, fitting, and the construction of accurate spectroscopic models. The complex topology of adiabatic representations makes the determined physics of the system sensitive to small variations in the topology of the property curves, which is undesirable for theoretical models. Therefore, the diabatic representation offers a simpler, more stable model, where the derived physics is less susceptible to variations in its property curves.

Equivalence between the adiabatic and diabatic representations in nuclear motion calculations is often assumed but seldom shown. For the nuclear motion Schrödinger equation, the solution should be independent of the chosen representation [16], as real observables are frame‐independent. This claim of course only holds when considering a single nuclear coordinate (i.e., for a diatomic, which this study concerns), where a strict diabatic representation can be found. For more nuclear degrees of freedom, an exactly equivalent diabatic representation cannot be constructed in general, instead it is common to build a quasi‐diabatization where the NAC terms are minimised. The only documented comparison between the adiabatic and diabatic representations for nuclear motion calculations was done recently by Brady et al. [29]. Their study demonstrated numerical equivalence for a two‐state coupled electronic system, specifically for the rovibronic energies of the 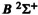 and 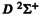 states of YO, and the 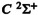 and 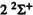 states of CH. It was also shown that there is no one choice of representation to use, but depends on the system studied, in particular the topology of the avoided crossing. For numerical applications, the precision of the computed observables will increase with increasing accuracy of the calculation, such as with basis size, and it was shown in the same study [29] that convergence rates for the vibronic energies was faster diabatically for YO but initially faster adiabatically for CH, an example that the choice of frame is important for physical problems. Other than this, convergence between the adiabatic and diabatic states has been investigated by few papers. The adiabatic and diabatic states of the transition probability amplitudes in collisions of collinear atom–diatom systems have been shown to be equivalent by Zimmerman and George [30], where the diabatic representation converged significantly faster. Shi et al. [31] demonstrated equivalence and numerical convergence rates for the sinc‐DVR method in determining adiabatic and diabatic energy eigenvalues and eigenfunctions but required using a complete adiabatic model and a conical intersection at high energy. Despite no other direct comparisons being found in the literature, the series of papers by Wolniewicz, Dressler and co‐workers [32, 33, 34, 35, 36, 37, 38] illustrated the importance of the DDR couplings and effectiveness of the diabatic representation for the rovibronic energy calculations of molecular hydrogen's excited electronic states. It was shown that NACs played an important role for the production of an accurately computed spectroscopy of the system, where in the later studies the diabatic representation was also shown to provide an accurate description of the nuclear dynamics of ${\text{H}}_{2}$. Additionally, Pachucki and Komasa [39, 40] developed a nonadiabatic perturbation theory to incorporate NAC terms in their rovibronic treatment of ${\text{H}}_{2}$. They demonstrated that inclusion of these perturbative corrections yielded accurate rovibronic energy terms. Further demonstrating the importance of NAC terms on the computed rovibronic solution of diatomic molecules.

Nonadiabatic interactions, the associated DDRs, and the adiabatic‐diabatic equivalency is important in many applications other than rovibronic calculations. In particular, the assumption of adiabatic‐diabatic equivalence has been crucial for scattering calculations. For example, an adiabatic and diabatic reformulation of the mutual neutralization reaction ${\text{H}}^{+}+{\text{H}}^{-}\to {\text{H}}_{2}^{*}\to \text{H}(1)+\text{H}(n)$ (where n=1,2,3, etc. is the principal quantum number) by Volkov et al. [41] produced cross‐sections consistent with other competing methods, and demonstrated equivalent results between the adiabatic and diabatic frames. It was further shown that the second DDR term had a significant impact on the accurate computation of the cross‐sections. Furthermore, a partial diabatic representation for the ${\text{N}}_{2}$ electronic structure was made by Little and Tennyson [42] in order to complete multichannel quantum defect theory calculations for the dissociative recombination of ${\text{N}}_{2}^{+}$ [43]. These examples confirm the need for accurate representation of nonadiabatic dynamics in general.

We demonstrate, for the first time, numerical equivalence between the adiabatic and diabatic representations for multi‐electronic‐state coupled systems of diatomic molecules by direct application to a 3‐state ${\text{N}}_{2}$ ab initio system, 4‐state CH ab initio system, and an artificially generated 10‐state system (with PECs illustrated in Figure 1). To this end, a new nonadiabatic rovibronic module the duo code is implemented. For the benchmarks on CH and ${\text{N}}_{2}$, recently published ab initio curves are used Gelfand et al. [44], Brady [45]. The importance of nonadiabatic coupling in these highly correlated systems are then investigated, where a complete description of the complex nonadiabatic interactions and PECs of these molecules will be valuable to many fields. For example, CH is one of the most extensively studied free radicals [46] due to its presence in a wide range of environments. Astrophysically, it has been detected in both solar [47, 48, 49] and stellar spectra [50, 51, 52], in the spectra of comets [53], the interstellar medium (ISM) [54, 55, 56, 57], and molecular clouds [58]. ${\text{N}}_{2}$ is also an important molecule since it makes up nearly 78 percent of Earth's atmosphere. ${\text{N}}_{2}$ has also been observed within our solar system in the UV [59, 60, 61] and the ISM [62]. Lastly, highly excited electronic states of simple diatomics exhibit complex nonadiabatic behaviour and complex electronic structure [63], making the understanding of many coupled state systems and their interactions important. For example molecules like ${\text{C}}_{2}$, CN, ${\text{N}}_{2}$, SiC, ${\text{Si}}_{2}$, ${\text{O}}_{2}$, NO and their corresponding ions [63, 64, 65] exhibit these effects and motivates this study.

The selection of CH and ${\text{N}}_{2}$ for construction of model 4‐state and 3‐state benchmarks for this study is motivated by the contrasting extremes of their nonadiabatic behaviour. $N_{2}$ is characterized by strong, sharp NACs and avoided crossings between clearly bound PECs near their potential minima. In contrast, CH exhibits weak, wide NACs and broad avoided crossings near dissociation. Thus, we do not aim to provide empirically accurate data for these specific molecules, but use them as representative systems for numerical equivalence tests of nonadiabatic effects in diatomic molecules.

## 2 Theory

### 2.1 Diabatisation of the N‐State Coupled Diatomic System

We start with a pure vibrational nuclear motion Schrödinger equation for a diatomic molecular in the adiabatic representation as given by

$$H^{\left(a\right)}\vec{\varphi }(r)=E\vec{\varphi }(r)$$

where r is the internuclear distance, $\vec{\varphi }(r)$ is a vector of vibronic wavefunctions of size N, E are the vibronic energies, and $H^{\left(a\right)}$ is the adiabatic, pure vibronic, Hamiltonian matrix. Within the adiabatic representation, the nuclear kinetic energy matrix contains diagonal and off‐diagonal derivative couplings (DDRs) which couple electronic and nuclear motion and are neglected in the BO approximation. The DDRs couple electronic states of the same symmetry [6, 8, 16, 18, 19, 66] and form cusp‐like curves near the regions of avoiding crossings between the state PECs. These nonadiabatic interactions are illustrated via the following Born‐Huang N×N Hamiltonian matrix [18, 19, 29, 66]

(1) $$\begin{array}{l} H^{\left(a\right)}=-\frac{\hbar^{2}}{2\mu }\left(\vec{\frac{d^{2}}{dr^{2}}}-K-\left[\overset{\leftarrow }{\frac{d}{dr}}W^{\left(1\right)}-W^{\left(1\right)}\vec{\frac{d}{dr}}\right]\right)+V^{\left(a\right)} \end{array}$$

The directions of the derivatives are shown and is how we program the kinetic energy operator in our rovibronic code duo (see Section 2.2). μ is the reduced mass of the diatomic molecule, $V^{\left(a\right)}$ is the adiabatic (diagonal) electronic potential matrix with elements $V_{ii}^{\left(a\right)}(r)$ being the PECs, $W^{\left(1\right)}$ is the skew‐symmetric NAC matrix with elements being the first DDRs couplings $W_{ij}^{\left(1\right)}$ given by

(2) $$W_{ij}^{\left(1\right)}=\left\{\begin{array}{c} \left\langle \psi_{i}^{\text{a}}\right|\frac{d}{dr}\left|\psi_{j}^{\text{a}}\right\rangle =-\left\langle \psi_{j}^{\text{a}}\right|\frac{d}{dr}\left|\psi_{i}^{\text{a}}\right\rangle ,\;\;i\neq j\;\\ 0,\;\;i=j\; \end{array}\right.$$

Lastly, K is then the second DDR term with matrix elements

(3) $$K_{i,j}=\left\langle \frac{d\psi_{i}^{a}}{dr}|\frac{d\psi_{j}^{a}}{dr}\right\rangle$$

with diagonal elements when multiplied by the kinetic energy factor, $-\frac{\hbar^{2}}{2\mu }K_{\rho \rho }$, give the well‐known DBOCs, and its off‐diagonal elements form further second DDR couplings for systems of three or more states. The K matrix can be trivially computed via the squared NAC matrix as

(4) $$K=-W^{\left(1\right)}\cdot W^{\left(1\right)}$$

Diagonalisation of the kinetic energy, thereby removing simultaneously the first and second DDR couplings together with the DBOC, was shown to be possible by Mead and Truhlar [22]. The representation where the nuclear kinetic energy is diagonal is known as the diabatic representation, and transformation to this representation can be achieved by action of a unitary transformation (the AtDT) on the adiabatic Hamiltonian. This r‐dependent unitary transformation mixes the adiabatic electronic wavefunctions to yield diabatic states via

(5) $$\psi_{j}^{\left(d\right)}(\xi ;r)=\overset{N}{\underset{i=1}{\sum }}U_{ij}(r)\psi_{i}^{\left(a\right)}(\xi ;r)$$

where ξ are the electronic coordinates. To ensure the radial DDR terms are removed, it is required that the derivatives of the diabatic electronic states with respect to the nuclear coordinate, r, be zero (or negligible). After this transformation, the radial DDR terms are eliminated, and the nuclear kinetic energy is diagonalized. To find the required AtDT matrix, solution to the following first order differential equation is required [16, 67, 68]

(6) $$\frac{dU}{dr}=-W^{\left(1\right)}U$$

where $W^{\left(1\right)}$ is the NAC matrix with elements given by Equation (2). Solutions to Equation (6) have been studied in the literature [16, 19, 67]. In this work, we adopt the diabatisation scheme recently developed by Brady [45], in which the AtDT is computed via an evolution method, guided by the NAC terms. The resulting transformation is then regularised to achieve internal consistency between the NAC elements and the adiabatic potentials.

Once U is determined, the diabatic Hamiltonian can be found by transforming Equation (1) as so

(7) $$\begin{array}{l} H^{\left(d\right)}=U^{†}H^{\left(a\right)}U=-\frac{\hbar^{2}}{2\mu }\vec{\frac{d^{2}}{dr^{2}}}+V^{\left(d\right)} \end{array}$$

where the nuclear kinetic energy matrix is diagonalised with no DDR coupling at the cost of introducing DCs into the diabatic potential matrix, $V^{\left(d\right)}$, with elements

(8) $$V_{ij}^{\left(d\right)}=\left\{\begin{array}{c} d_{ij},\;\;i\neq j\;\\ V_{i}^{\left(d\right)},\;\;i=j\; \end{array}\right.$$

Here, $d_{ij}$ are the DCs which couple different electronic states.

### 2.2 Solving the Rovibronic Schrödinger Equation

To test the equivalence between adiabatic and diabatic representations of multistate coupled diatomic systems, and hence prove a numerically strict diabatic basis can been found, a complete rovibronic solution to the diatomic problem is accomplished by extension of the pure vibrational Hamiltonian operator in Equations (1) or (7) with the rotation‐spin‐electronic contribution as follows (see Yurchenko et al. [69] for details of the approach used):

(9) $$Ĥ={Ĥ}_{\text{vib}}+\frac{\hbar^{2}}{2\mu }{\hat{R}}^{2}$$

where the rotational angular momentum operator $\hat{R}$ is now given by

(10) $$\hat{R}=Ĵ-Ŝ-\hat{L}$$

Here, Ĵ, Ŝ, $\hat{L}$ are the total, spin, and orbital electronic angular momenta, respectively. In our current treatment, we do not couple the nuclear spin and therefore do not consider hyperfine effects. We then solve the adiabatic and diabatic rovibronic Schrödinger systems variationally on the Hund's case (a) basis using the duo program [69], including all nonadiabatic effects. To solve the Schrödinger equation for curves defined on either grid or analytic representations, duo uses the numerical sinc‐DVR method [70, 71, 72]. For a grid input, like the spectroscopic models presented here, natural cubic splines [73] are used to map the grid onto sinc‐DVR points for all curves. The DBOC terms can be either provided as input or generated from the NAC using Equation (4).

It was shown by Brady et al. [29] that numerical equivalence between the adiabatic and diabatic representations within nuclear motion calculations is possible for the coupled two‐state case, even subject to the convergence (because of PEC‐adapted vibrational basis set) or other numerical limitations. It was also shown that neither the adiabatic or diabatic model is generally better, but depends on the system studied. However, the coupled two‐state system is an approximation, which can be justified by Hellman‐Feynman theorem [74, 75, 76, 77, 78, 79], to the real system of an infinite number of coupled adiabatic states. In this work, we demonstrate the effect of couplings to higher energy states on the rovibronic energies, and hence wavefunctions.

To test the importance of different coupling terms for nuclear motion calculations, different approximations to the adiabatic and diabatic representations are made and the resulting energy terms are compared. We consider four approximations to the rovibronic solution for the different molecular systems treated here: I, the case when DBOCs are omitted from the adiabatic representation; II, the case when all off‐diagonal DDR couplings are omitted ($K_{i\neq j}=W_{ij}^{\left(1\right)}=0$) from the adiabatic representation; III, the case when all DDR couplings (DBOC, off‐diagonal DDRs) are omitted from the adiabatic representation; IV, the case when DCs are omitted from the diabatic representation. It will be demonstrated in the following sections, and should be expected, that these approximations should result in a significant impact on the spectroscopy of the studied system and as a result the quantum number labelling may become noncomparative between the different approximations. We choose to study a combination of the energy enumeration, n, quantum number labelling, and character of the (“vibronic wavefunction”) reduced density state (see Brady et al. [29] for more details), where only the closest matching states will have their energy compared. It has been discussed that state numbering leads to accurate assignments of the bound rovibronic levels, as summarised by the oscillation theorem [80, 81] which states that *the* $i^{\text{th}}$ *bound rovibronic eigenfunction has* i *internal nodes*, but breaks down in the strongly coupled case where the single‐state approximation is no longer valid.

## 3 Spectroscopic Models

To demonstrate numerical equivalence of the adiabatic and diabatic representations within nuclear motion calculations for the general N‐state diatomic problem, we begin by demonstrating this equivalence using an extreme synthetic system. Following this, we apply physical spectroscopic models of real molecular systems with fewer interacting states to highlight the importance of various nonadiabatic coupling terms, including diabatic couplings. Application to these real molecular systems then serves as a benchmark for future studies on similar systems. Finally, the synthetic 10‐state system is revisited to explore the effect of truncating the number of adiabatic states on the computed rovibronic energy levels.

### 3.1 The 10‐State Solution

The extension to N‐state coupled diatomic systems should have no limit to the number of states treated, N. It is interesting to test adiabatic‐diabatic equivalence on a large system, for example a 10‐state problem, as a demonstration of the robustness of the used diabatization method and exactness of the AtDT. We present a synthetic molecule with 10 coupled states, similar to the high‐energy systems of molecules such as ${\text{C}}_{2}$, CN, ${\text{N}}_{2}$, SiC, and ${\text{Si}}_{2}$, all of which exhibit a complex network of adiabatic avoided crossings and a single deep diabatic potential well that spans the entire potential landscape up to high dissociation limits [64, 65]. This model was taken from the recent study by Brady [45].

Figure 1 illustrates the synthetically generated 10‐state coupled diatomic system in the 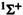 electronic manifold, where the NACs and subsequently the diabatic representation were generated using the hybrid asymptotic‐property‐based diabatization method of Brady [45]. The 45 NACs were regularised after guessing them in the initial generation of the adiabatic model, showing the robustness of the used regularisation scheme [45].

The lowest 1000 J=0 rovibronic energies for the fully coupled 10‐state system were computed in the adiabatic and diabatic representations. Table 1 lists selected rovibronic energy terms chosen to be situated near and avoided crossings. Despite the large correlation in this highly coupled system, equivalence has been shown between the adiabatic and diabatic representations where a numerically exact AtDT has been found. Table 1 shows the adiabatic and diabatic rovibronic energies to agree at least to within $10^{-6}$ cm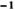. This shows that one can model large systems for an arbitrary number of states in duo without loss of strictness in the diabatic basis.

**TABLE 1:** Selected J=0 rovibronic energy term values (in cm) of the synthetic 10 coupled state state system computed within the adiabatic and diabatic representations using duo.

n	Adiabatic				Diabatic	
	$\tilde{E}$	State	v	$\tilde{E}$	State	v
7	1847.007771	$1^{1}\sum^{+}$	6	1847.007771	$2^{1}\sum^{+}$	3
8	2129.925260	$1^{1}\sum^{+}$	7	2129.925261	$2^{1}\sum^{+}$	4
9	2416.431857	$1^{1}\sum^{+}$	8	2416.431858	$2^{1}\sum^{+}$	5
30	6678.428517	$2^{1}\sum^{+}$	3	6678.428518	$3^{1}\sum^{+}$	0
32	6984.594060	$2^{1}\sum^{+}$	4	6984.594060	$3^{1}\sum^{+}$	1
35	7298.846922	$2^{1}\sum^{+}$	5	7298.846922	$3^{1}\sum^{+}$	2
114	12464.895630	$1^{1}\sum^{+}$	84	12464.895630	$1^{1}\sum^{+}$	13
115	12523.517670	$3^{1}\sum^{+}$	5	12523.517670	$2^{1}\sum^{+}$	80
116	12586.520910	$2^{1}\sum^{+}$	24	12586.520910	$3^{1}\sum^{+}$	20
247	18814.479740	$3^{1}\sum^{+}$	26	18814.479740	$4^{1}\sum^{+}$	21
249	18895.065350	$5^{1}\sum^{+}$	0	18895.065350	$5^{1}\sum^{+}$	0
251	18981.836630	$3^{1}\sum^{+}$	27	18981.836620	$4^{1}\sum^{+}$	22
253	19032.279350	$1^{1}\sum^{+}$	127	19032.279350	$1^{1}\sum^{+}$	21
426	25580.915070	$5^{1}\sum^{+}$	11	25580.915070	$6^{1}\sum^{+}$	2
427	25596.980210	$6^{1}\sum^{+}$	2	25596.980210	$6^{1}\sum^{+}$	3
435	25915.997070	$4^{1}\sum^{+}$	32	25915.997070	$5^{1}\sum^{+}$	27
662	33094.870560	$6^{1}\sum^{+}$	18	33094.870560	$5^{1}\sum^{+}$	98
663	33124.269800	$5^{1}\sum^{+}$	43	33124.269800	$3^{1}\sum^{+}$	160
664	33161.059010	$2^{1}\sum^{+}$	164	33161.059010	$6^{1}\sum^{+}$	37
669	33265.317660	$7^{1}\sum^{+}$	7	33265.317660	$7^{1}\sum^{+}$	8
949	40738.198500	$6^{1}\sum^{+}$	70	40738.198500	$7^{1}\sum^{+}$	62
950	40787.878880	$7^{1}\sum^{+}$	25	40787.878880	$1^{1}\sum^{+}$	54
958	40985.704660	$8^{1}\sum^{+}$	13	40985.704660	$6^{1}\sum^{+}$	110
1267	48624.059359	$9^{1}\sum^{+}$	20	48624.059359	$8^{1}\sum^{+}$	80
1270	48668.417006	$5^{1}\sum^{+}$	154	48668.417006	$3^{1}\sum^{+}$	217
1271	48684.060547	$1^{1}\sum^{+}$	241	48684.060547	$2^{1}\sum^{+}$	236
1272	48743.879213	$7^{1}\sum^{+}$	90	48743.879213	$8^{1}\sum^{+}$	81
1273	48769.629102	$3^{1}\sum^{+}$	202	48769.629102	$4^{1}\sum^{+}$	197
1276	48833.738010	$10^{1}\sum^{+}$	0	48833.738010	$10^{1}\sum^{+}$	0
1626	56637.515727	$10^{1}\sum^{+}$	32	56637.515727	$10^{1}\sum^{+}$	32
1627	56656.811037	$8^{1}\sum^{+}$	105	56656.811037	$9^{1}\sum^{+}$	96
1629	56677.102187	$7^{1}\sum^{+}$	138	56677.102187	$8^{1}\sum^{+}$	130
1648	57082.586039	$4^{1}\sum^{+}$	208	57082.586039	$5^{1}\sum^{+}$	205
1669	57491.954867	$9^{1}\sum^{+}$	61	57491.954867	$10^{1}\sum^{+}$	43
	…			…		

*Note:* These states were chosen to be situated at the avoided crossing regions.

### 3.2 The ${\text{N}}_{2}$ Solution

Numerous studies have demonstrated that molecular nitrogen possesses a complex electronic structure [44, 82, 83], particularly highlighting the avoided crossings between the 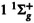, 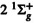, 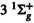 states, which are strongly bound and exhibit significant nonadiabatic coupling. The studied ${\text{N}}_{2}$ model then serves as a benchmark for systems with strong NAC near the potential minima. For this study, the PECs and NACs of the [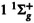, 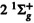, 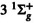] system were sourced from the work of Gelfand et al. [44], who carried out ab initio calculations in molpro at the multireference configuration interaction (MRCI) level of theory. These computations employed aug‐cc‐pVQZ basis sets, with the molecular orbitals optimized in preliminary complete active space self‐consistent field (CASSCF) calculations. The PECs were further extended to higher dissociation limits and the NACs were regularized to maintain internal consistency with the adiabatic potentials following the method outlined in Brady [45]. This is the model we adopt in our rovibronic treatment of ${\text{N}}_{2}$.

**FIGURE 2:**
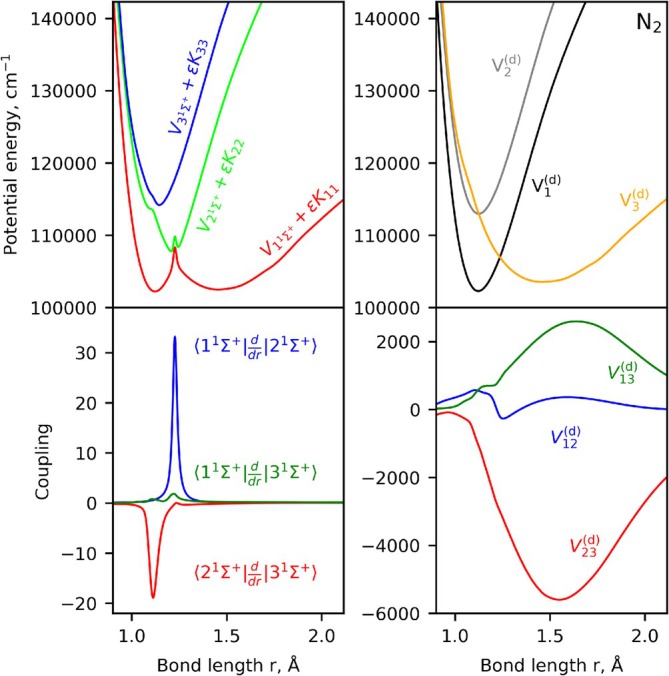
Illustration of the diabatization of the ${\text{N}}_{2}$ [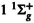, 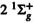, 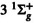] system: Adiabatic PECs (top left), NACs (bottom left), diabatic PECs (top right), and DCs (bottom right). The DBOC corrections have been added to the adiabatic potentials and are computed from multiplying the kinetic energy factor $\epsilon =\frac{\hbar }{8\pi^{2}\mu c}$ by the diagonal elements of the K matrix.

The adiabatic ${\text{N}}_{2}$ model is diabatized using the hybrid asymptotic‐property‐based diabatization method detailed in Brady [45], where solution to Equation (6) is found and ensures physical asymptotic behavior and smoothness of the resulting diabatic properties. The adiabatic and diabatic representation of the ${\text{N}}_{2}$ [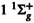, 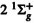, 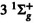] system we treat rovibronically are illustrated in Figure 2. The DBOC coupling $K_{\rho \rho }$ is added to the adiabatic PECs for clarity and illustrates the significant difference between the adiabatic and diabatic models—a large spike in the middle of the adiabatic PECs and a smooth set of diabatic PECs. Still, we expect the two models to produce the same eigenvalues and eigenfunctions.

The lowest 37 rovibronic energy term values (J=0) computed using the adiabatic and diabatic models are listed in Table 2. While the approximate quantum state numbers are very different between the adiabatic and diabatic representations, the state energies are identical to within $4\times 10^{-8}$ cm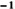. duo assigns quantum labels via the largest contribution from the corresponding basis sets, which in both cases are very different and so are their state interpretations, in which case we compare states of matching energy enumeration. Comparison of the adiabatic and diabatic reduced density states reveals that their wavefunctions are identical, confirming the comparison of rovibronic energies with the same energy enumeration is correct. In approximate cases, quantum numbers and energy enumeration fail as reliable state labels, requiring inspection of reduced density states for meaningful comparison with fully coupled cases. This inspection is done both visually and via studying the Euclidean distance between two reduced densities (see Equation 11 below). The approximate solutions include many nonphysical intermediate states (denoted by dots in the table). In some extreme cases, state assignment is too ambiguous for comparison. In some cases, highlighted in Table 2 in bold, only partial match of the radial densities can be established between approximate and fully coupled solutions.

**TABLE 2:** The lowest 36 J=0 rovibronic energy term values (cm) of the ,  and  states of $N_{2}$ computed within the adiabatic and diabatic representations using duo (columns labeled with E).

n	Adiabatic						Diabatic			
	E	E(I)	E(II)	E(III)	State	v	E	E(IV)	State	v
1	0.000000	0.00	0.00	0.00	1	0	0.000000	491.13	3	0
2	457.775275	434.70	467.73	449.72	1	1	457.775275	0.00	1	0
3	693.564594	692.31	694.21	693.35	1	2	693.564594	1147.15	3	1
4	1416.011696	1405.99	1419.42	1412.73	1	3	1416.011696	1841.29	3	2
5	2131.061586	2063.32	2141.84	2102.13	1	4	2131.061586	2531.37	3	3
6	2537.890864	2343.14	2607.76	2464.90	1	5	2537.890864	2135.10	1	1
7	2868.126501	2816.77	2917.16	2907.74	1	6	2868.126501	3211.12	3	4
8	3549.801719	3411.32	3621.01	3561.95	1	7	3549.801719	3887.93	3	5
9	4210.218745	4012.17	4323.41	4182.42	1	8	4210.218745	4576.05	3	6
10	4695.005744	4466.69	4889.47	4757.11	1	9	4695.005744	4220.68	1	2
11	5133.725681	4916.04	5394.71	5374.48	1	10	5133.725681	5276.23	3	7
12	5790.155876	5622.04	6086.62	6042.51	1	11	5790.155876	5974.59	3	8
13	6479.408694	…	6814.52	…	1	12	6479.408694	6668.76	3	9
14	6779.063208	6312.32	…	…	2	0	6779.063208	6272.45	1	3
15	7213.410603	7161.51	7480.06	7376.44	1	13	7213.410603	7357.07	3	10
16	7903.009725	7866.38	8070.55	8027.39	1	14	7903.009725	8032.03	3	11
17	8583.091028	8515.76	8701.90	8681.31	1	15	8583.091028	8695.77	3	12
18	8881.592989	8770.03	…	…	2	1	8881.592989	8297.31	1	4
19	9252.763917	9147.87	9381.77	9335.86	1	16	9252.763917	9349.30	3	13
20	9904.981173	9794.81	10057.13	9984.60	1	17	9904.981173	9994.94	3	14
21	10539.639862	10440.51	10692.74	10624.65	1	18	10539.639862	10629.69	3	15
22	10877.912402	10683.08	…	…	2	2	10877.912402	10289.06	1	5
23	11141.610134	11029.36	11292.15	1	19	11141.610134	11254.07	3	16	
24	11664.870261	11932.75	…	…	1	20	11664.870261	…	2	0
25	12082.377293	11479.03	…	…	3	0	12082.377293	11867.94	3	17
26	12541.173804	12441.24	12528.33	12499.51	1	21	12541.173804	12470.76	3	18
27	12881.431795	12750.50	…	…	1	22	12881.431795	12244.60	1	6
28	13129.244992	12993.68	**13154.46**	**13108.83**	2	3	13129.244992	13061.27	3	19
29	13562.828800	13491.25	…	13706.29	1	23	13562.828800	…	2	1
30	13934.090728	13852.42	…	…	1	24	13934.090728	13640.03	3	20
31	14380.132252	14292.75	14335.15	14291.51	3	1	14380.132252	14205.53	3	21
32	14762.541917	14689.51	**14890.59**	14863.83	1	25	14762.541917	14205.53	1	7
33	14987.484472	14889.54	…	…	1	26	14987.484472	14757.42	3	22
34	15431.979951	15365.05	**15441.03**	15423.32	2	4	15431.979951	15294.68	3	23
35	15776.743132	15692.66	15988.98	…	1	27	15776.743132	15816.84	2	2
36	16094.153840	16038.83	…	15968.69	3	2	16094.153840	16322.97	3	24
	…	…	…	…			…	…		
$E_{\text{ZE}}$	102834.749359	102834.41	102834.81	102834.49			102834.749359	103364.63		
$E_{\text{rms}}$	n≤36	176.04	138.55	90.67				333.29		
$E_{\text{rms}}$	n≤100	680.15	407.77	420.26				492.62		
$\rho_{\text{rms}}$	n≤100	0.637000	1.434000	1.441000				0.755000		

*Note:* Varying degrees of approximations are used and are labeled: I is the case when DBOCs are omitted from the adiabatic representation; II is the case when all off‐diagonal DDR couplings are omitted ($K_{i\neq j}=W_{ij}^{\left(1\right)}=0$) from the adiabatic representation; III is the case when all DDR couplings (DBOC, off‐diagonal DDRs) are omitted from the adiabatic representation; IV is the case when DCs are omitted from the diabatic representation. All energies are relative to the energies of the corresponding v=0, 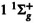 state, defined as the zero‐of‐energies ($E_{\text{ZE}}$) given at the bottom of each column. The bold numbers refer to states with difficult quantum number assignment (see text). The root mean square difference of the lowest n approximate and fully coupled rovibronic terms are computed for the energy ($E_{\text{rms}}$) and radial reduced densities ($\rho_{\text{rms}}$, see Equation (11)).

Now that numerical equivalence has been demonstrated for the three‐state problem, we can assess the significance of nonadiabatic coupling terms in the ${\text{N}}_{2}$ model. Table 2 also lists the rovibronic energies computed using approximations I, II, III, and IV (as described in Section 2.2). For the lowest energy levels, the exclusion of DCs (approximation IV) play a critical role in maintaining model accuracy. Omitting these couplings leads to significant discrepancies, with a root mean square error (RMSE) of 333.3 cm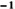 compared to RMSE values for the adiabatic approximations: RMSE(I) = 176.0cm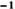, RMSE(II) = 138.6 cm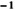, RMSE(III) = 90.7 cm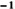.

**FIGURE 3:**
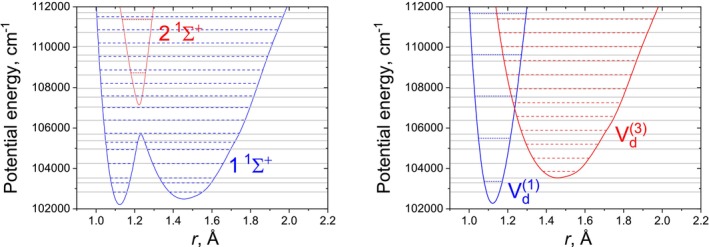
Illustration of the energy term values of our ${\text{N}}_{2}$ model (J=0) system computed using the adiabatic approximation III (dashed, left panel) and diabatic approximation IV (dashed, right panel) compared to the corresponding “exact” solution (no approximations, solid gray lines, both panels) for the lowest 18 states.

This large discrepancy is primarily due to the unexpectedly strong DCs in this strongly nonadiabatic coupled system, which contradict the predictions of Brady et al. [29], who anticipated smaller DCs when NACs are large for the two state case, similar to what was observed for YO. The substantial differences between the adiabatic and diabatic potential minima lead to a swapping of the ground and first excited states (as seen for n=1 and n=2 in Table 2), introducing a systematic offset in the energy agreement. This effect is particularly relevant for the lowest energy levels, which are of significant spectroscopic importance and thus warrant careful analysis. Figure 3 illustrates how some of the approximations affect the computed energies of ${\text{N}}_{2}$ by comparing states approximated energies (adiabatic III and diabatic IV) to the “exact” values, that is, computed with all associated couplings. It is clear that both approximations lead to strong deviations from the “exact” values with the diabatic case especially affected by the absence of the diabatic coupling. It is interesting that despite the adiabatic zero‐order approximation appearing to provide a better, more physically intuitive solution than in the zero‐order diabatic approximation, the convergence of the diabatic solution is faster for the latter, diabatic case.

In the region of the avoided crossing (states n=10−15), neglecting NACs results in discrepancies comparable to those seen when the DCs are omitted, with energy differences from the fully coupled case on the order of $10^{2}$ cm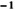. However, for states higher in energy than the crossing, the adiabatic approximations continue to break down as rovibronic energies deviate from the fully coupled case. When analysing the lowest 100 bound states, the RMSE increases significantly for adiabatic approximations (I, II, III), with the largest errors occurring when the DBOC is omitted. On the other hand, omitting the DCs result in a similar RMSE when more states are included. This suggests that the adiabatic representation for ${\text{N}}_{2}$ is less stable for highly excited states compared to the diabatic case.

Additionally, Table 2 highlights that the adiabatic approximation fails to capture certain states that are still present in the approximate diabatic case. Inspection of the reduced density states ($\rho_{i}$, see Eq. (24) of Brady et al. [29]) reveals that the wavefunctions in the adiabatic approximations struggle to reproduce the correct character seen in the fully coupled calculations. Deviation between the approximate and fully‐coupled reduced density states is quantified by their Euclidean distance, defined as

(11) $$\begin{array}{l} d(\rho_{i},\rho_{j})=\int {\left(\rho_{i}-\rho_{j}\right)}^{2}dr \end{array}$$

The RMSE for the Euclidean distance between the approximate and fully coupled reduced density states ($\rho_{\text{rms}}$) is twice as large for the adiabatic approximations than when the DCs are omitted from calculations. This indicates that wavefunctions computed in the approximate adiabatic representation differ significantly from those in the diabatic approximation IV, which may lead to inaccurate computed rovibronic intensities. Despite this, the induced errors via these approximations prove that for high‐resolution applications all nonadiabatic effects must be included. Performing convergence tests reveal that the ${\text{N}}_{2}$ energy terms in Table 2 have similar convergence rates with grid size between the adiabatic diabatic representation. However, Brady et al. [29] demonstrates that the diabatic representation yields significantly faster convergence with the size of the contracted rovibronic basis set than the corresponding adiabatic representation with strong NACs.

While this analysis suggests the adiabatic representation is more reliable for the lower lying states, with the diabatic representation being more stable for energetically higher states of the ${\text{N}}_{2}$ model, caution is needed when generalizing these results. The comparison here is specific to this model, and further complexities must be considered. Notably, the sensitivity of the spectroscopy in each representation is an important factor. Testing the effect of varying the NACs revealed that small changes in their magnitudes led to substantial variations in the DCs, on the order of $10^{4}$ cm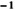. A reduction of NAC magnitudes by 20% resulted in a corresponding change of $10^{5}$ cm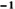 in the DCs. Therefore, while the DCs in this system are strong, the ${\text{N}}_{2}$ spectroscopy is likely to be less sensitive to the diabatic representation.

### 3.3 The CH Solution

**FIGURE 4:**
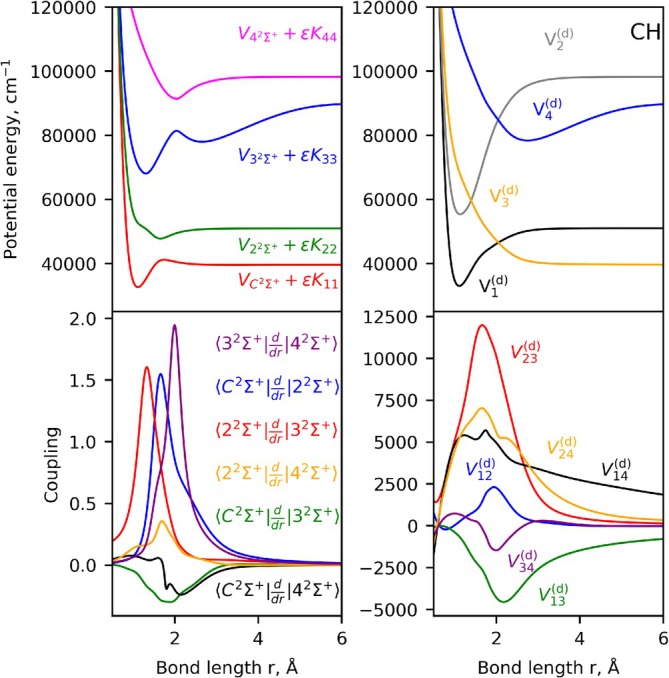
Illustration of the diabatization of the CH [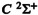, 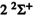, 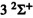, 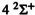] system: Adiabatic PECs (top left), NACs (bottom left), diabatic PECs (top right), and DCs (bottom right). The DBOC corrections have been added to the adiabatic potentials and are computed from multiplying the kinetic energy factor $\epsilon =\frac{\hbar }{8\pi^{2}\mu c}$ by the diagonal elements of the K matrix.

The work by van Dishoeck [84] and later by Kalemos et al. [85] provided ab initio calculations for highly excited electronic states of CH, where the [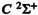, 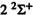] system (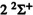 sometimes labeled 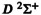) revealed a clear avoided crossing. The diabatization of this [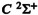, 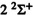] system has been investigated in depth by Brady et al. [29]. More recently, Brady [45] computed ab initio PECs for the four lowest doublet electronic states 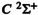, 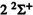, 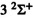 and 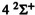 and the six NACs coupling these states using the quantum chemistry package molpro. Their calculations employed MRCI theory with weighted aug‐cc‐pwCVQZ basis sets on orbitals obtained from prior CASSCF calculations. In addition, Brady [45] regularized the ab initio NACs using a hybrid asymptotic‐property‐based diabatization method, which maximized internal consistency between the NACs and PECs. The diabatization for this four‐state coupled system was completed using the same method, Figure 4 illustrates the corresponding adiabatic (left panel, where the DBOC terms have been added to the adiabatic PECs) and diabatic (right panel) models which we adopt in our rovibronic treatment.

The CH [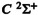, 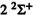, 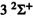, 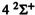] system is different to the studied ${\text{N}}_{2}$ [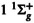, 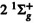, 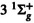] system in that, adiabatically, the CH PECs have large energetic separations, the NACs are weaker by an order of magnitude meaning no spike‐like contributions from the DBOC terms, and diabatically the DCs are an order of magnitude greater. Thus, the studied CH model serves as a benchmark for similar CH‐like systems with weak NAC, large energy separations, and broad avoided crossing structures. As described by Brady et al. [29], above the first CH dissociation channel (39220.0 cm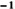) is heavily (pre‐)dissociated and contains (pre‐)dissociative and continuum states. These states are separated from this analysis by removing wavefunctions which oscillate at the “right” border $r_{\max}\to \infty$, which we assign as continuum states. Other states have the bound‐like character with their wavefunctions vanishing completely [86, 87]. It was shown in Brady et al. [29] that the continuum solution remains equivalent through diabatisation of the spectroscopic model, where both continuum wavefunctions and photo‐absorption spectra to these states are shown to be equivalent when computed using adiabatic and diabatic models. Therefore, only the “bound” state eigensolutions to the rovibronic Schrödinger equation for the CH problem will be studied. It should be noted that strictly speaking all states above the lowest dissociation are dissociative, including states selected here. Because of the couplings to the true‐dissociative states included in our model, they all exhibit some oscillatory behavior. In practice, threshold parameters are used to discriminate states with pronounced bound character from continuum states, for details see for example, Uhlikova et al. [88].

The lowest 17 bound J=0.5 e parity rovibronic energy levels computed using both the adiabatic and diabatic CH models are listed in Table 3. The energy values match to within $5\times 10^{-8}$ cm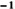 in both representations, confirming their equivalence for the 4‐state system. A strict diabatic basis for the CH system has been established, yielding results that are numerically equivalent to the adiabatic model as computed using the duo program. A comparison of rovibronic energies calculated via the approximate adiabatic and diabatic models indicates that the DCs play a critical role in ensuring model equivalence, both in terms of rovibronic energy and wavefunctions. Table 3 presents the RMSE for the studied bound state energies, showing that omitting the DCs results in a RMSE an order of magnitude greater than the approximate adiabatic calculations. Additionally, the approximate diabatic calculations yield significantly poorer reduced density states compared to the adiabatic approach, as reflected in the RMS of the Euclidean distance between the approximate and fully coupled reduced density states (see Equation 11). Thus, the approximate adiabatic representation more accurately reproduces the spectroscopy of the CH system than the approximate diabatic representation. This aligns with the conclusion of Brady et al. [29], who studied the numerical equivalence of the CH [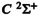, 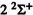] 2‐state system and found that weakly nonadiabatic systems generate large DCs, making the adiabatic representation a more appropriate framework for modeling the spectroscopy. However, the induced errors via these approximations prove that for high‐resolution applications all nonadiabatic effects must be included.

**TABLE 3:** The lowest 17 J=0.5 e parity rovibronic energies of the [, , ,] system of CH as computed within the adiabatic and diabatic representations using duo.

Adiabatic						Diabatic				
	$\tilde{E}$	$\tilde{E}$(I)	$\tilde{E}$(II)	$\tilde{E}$(III)	State	v	$\tilde{E}$	$\tilde{E}$(IV)	State	v
	0	0	0	0	1	0	0	0	1	0
	2603.377370	2601.61	2603.91	2602.15	1	1	2603.377370	2636.14	1	1
	4945.843448	4939.04	4947.94	4941.15	1	2	4945.843448	5061.21	1	2
	16789.795532	16778.98		16773.52	2	3	16789.795532		1	10
	35292.621456	35251.74	35286.78	35245.85	3	0	35292.621456		2	4
	44373.500709	44372.59	44373.46	44372.55	3	5	44373.500709	44052.40	4	0
	45130.519202	45128.91	45130.66	45129.04	3	6	45130.519202	44832.40	4	1
	45858.509178	45855.77	45858.90	45856.16	3	8	45858.509178	45585.66	4	2
	46548.411670	46542.70	46548.79	46543.00	3	9	46548.411670	46313.23	4	3
	47255.394177	47247.31	47253.94	47246.11	3	11	47255.394177	47016.19	4	4
	47841.236755	47829.32	47841.29	47829.25	3	12	47841.236755	47695.43	4	5
	48329.046921		48326.19	48306.66	3	13	48329.046921	48351.65	2	12
	48752.567999	48738.67	48749.41	48735.75	3	14	48752.567999	48351.65	4	6
	49246.485508	49236.12	49245.62	49235.33	3	15	49246.485508	48985.97	4	7
	49754.863246	49743.51	49754.64	49743.26	3	16	49754.863246	49599.12	4	8
	50243.670156	50232.21	50243.28	50231.83	3	17	50243.670156	50188.72	4	9
	50724.086185	50714.07	50723.88	50713.92	3	18	50724.086185	50753.76	4	10
	…	…	…	…			…	…		
$E_{\text{ZE}}$	33961.631668	33960.58	33961.76	33960.71			33961.631668	34430.76		
$E_{\text{rms}}$	13.11	1.94	15.40				191.07			
$\rho_{\text{rms}}$	0.000919	0.000200	0.002045				0.516103			

*Note:* Varying degrees of approximations are used and are labeled: I is the case when DBOCs are omitted from the adiabatic representation; II is the case when all off‐diagonal DDR couplings are omitted ($K_{i\neq j}=W_{ij}^{\left(1\right)}=0$) from the adiabatic representation; III is the case when all DDR couplings (DBOC, off‐diagonal DDRs) are omitted from the adiabatic representation; IV is the case when DCs are omitted from the diabatic representation. All energies are relative to the energies of the corresponding v=0, 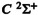 state, defined as the zero‐of‐energies ($E_{\text{ZE}}$) given at the bottom of each column. The root mean square difference of the approximate and fully coupled rovibronic terms are computed for the energy ($E_{\text{rms}}$) and radial reduced densities ($\rho_{\text{rms}}$, see Equation 11).

**FIGURE 5:**
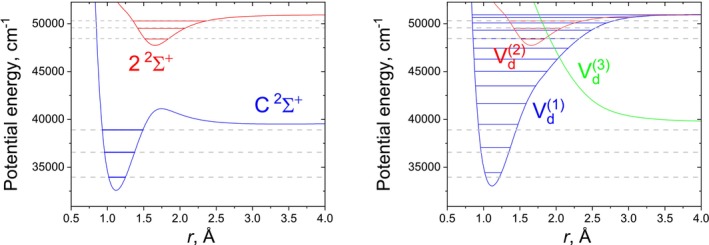
Illustration of the energy term values of our CH model system (J=0.5, e) computed using the adiabatic approximation III (dashed, left panel) and diabatic approximation IV (dashed, right panel) compared to the corresponding “exact” solution (no approximations, solid gray lines, both panels) for the lowest 6 states.

We now graphically illustrate the impact of these approximations on the energies of our model CH system in Figure 5 by plotting the states corresponding to the adiabatic approximation III and diabatic approximations IV and comparing them to the corresponding “exact” values (no approximation). While the adiabatic system of CH appears to be relatively immune for the absence of the couplings here, the diabatic zero‐order approximation has a dramatic effect on the positions and even physical meaning of the computed states. Indeed, not only the approximated energies are very different, the very steep potential well of $V_{1}^{\left(d\right)}$, when not connected to the repulsive state $V_{3}^{\left(d\right)}$, yields additional bound states in the region above the adiabatic dissociation of the 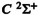 state.

### 3.4 Validity of the Two‐State Approximation

The two‐state approximation refers to the coupling of only two adiabatic states, meaning the K matrix in Equation (3) is diagonal and the DBOCs are given by the NAC squared between states 1 and 2 [29]. The two‐state approximation is attractive since solution to Equation (6) is analytic, and hence a diabatic representation is exactly known, modeling of the full adiabatic or diabatic system is simple and requires only parameterization of the NAC and two simple Morse and/or repulsive curves [29]. The argument for such an approximation can be made via Hellman–Feynman theorem, which relates the difference in adiabatic energies to the NAC via (see references [16, 89, 90] for details)

(12) $$\begin{array}{l} W_{\alpha ,\beta }^{\left(1\right)}=\frac{1}{E_{\beta }-E_{\alpha }}\left\langle \psi_{\alpha }^{a}|\frac{d{Ĥ}^{\left(a\right)}}{dr}|\psi_{\beta }^{a}\right\rangle \end{array}$$

Hence, if states α and β are sufficiently well seperated, that is, $|E_{\beta }-E_{\alpha }|\gg 1$, then the DDR matrix elements are small $W_{\alpha ,\beta }^{\left(1\right)}\ll 1$.

The DBOC $K_{\alpha ,\beta }=\left\langle \frac{d\psi_{\alpha }^{a}}{dr}|\frac{d\psi_{\beta }^{a}}{dr}\right\rangle$ can then be expressed in terms of the energy seperation as follows

(13) $$\begin{array}{ll} K_{\alpha ,\beta } & =\overset{N}{\underset{\kappa }{\sum }}\frac{1}{\left(E_{\alpha }-E_{\kappa })(E_{\kappa }-E_{\beta }\right)}\left\langle \psi_{\alpha }^{a}|\frac{d{Ĥ}^{\left(a\right)}}{dr}|\psi_{\kappa }^{a}\right\rangle \left\langle \psi_{\kappa }^{a}|\frac{d{Ĥ}^{\left(a\right)}}{dr}|\psi_{\beta }^{a}\right\rangle \; \end{array}$$

where the summation is over all adiabatic states and in the last line we inserted the Hellman–Feynman relation in Equation (12). We see that for the coupled two‐electronic state system, states $\left|\psi_{1}^{a}\right\rangle$ and $\left|\psi_{2}^{a}\right\rangle$, the NAC elements coupling other states will be reduced by a factor of $\frac{1}{\left(E_{\rho }-E_{\kappa })(E_{\kappa }-E_{\rho }\right)}$, for sufficiently well separated states from the coupled system $\left|\psi_{1}^{a}\right\rangle$ and $\left|\psi_{2}^{a}\right\rangle$ the summation is truncated yielding

(14) $$\begin{array}{l} K_{\rho ,\rho }\approx -{\left(W_{1,2}^{\left(1\right)}\right)}^{2} \end{array}$$

**FIGURE 6:**
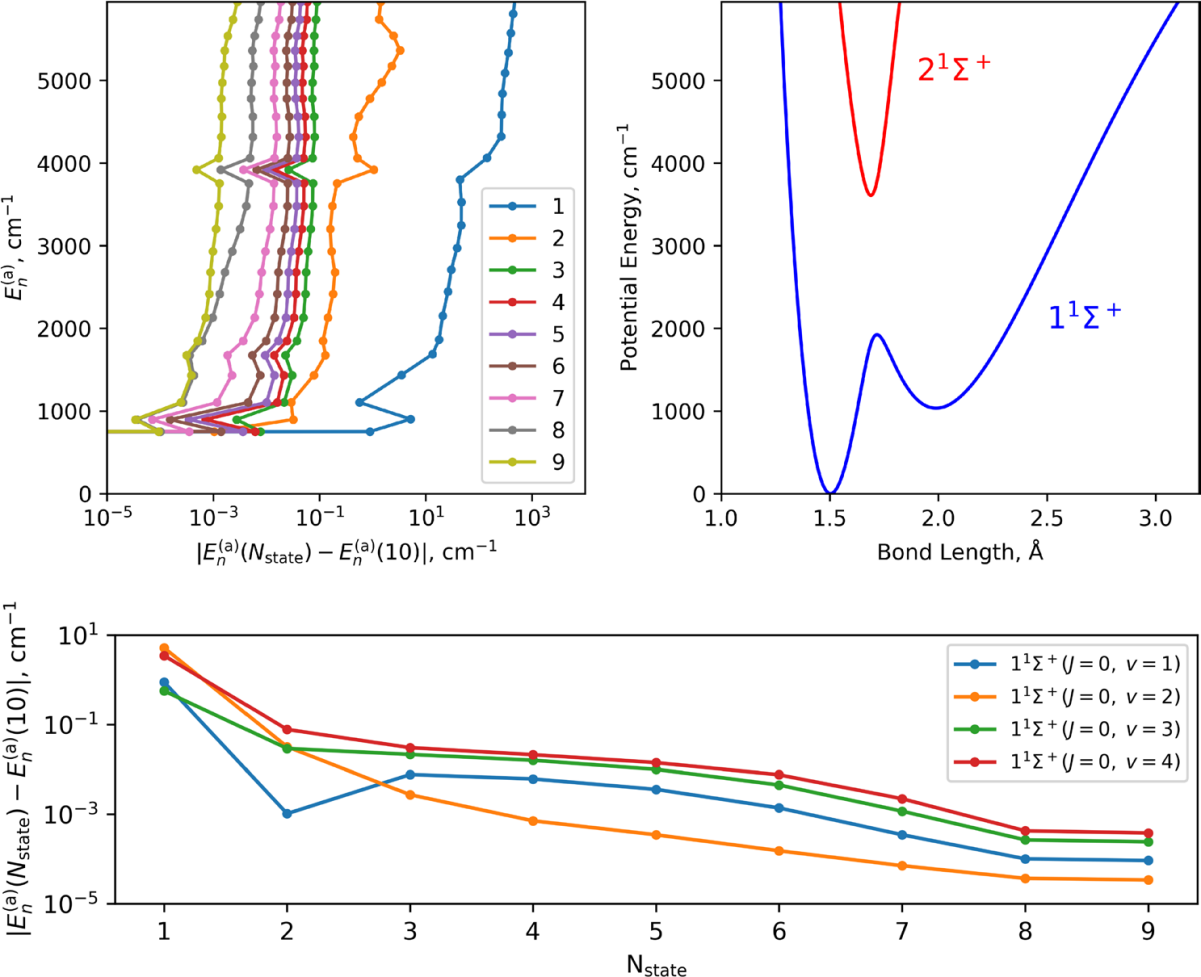
Illustration of the difference between the lowest 25 adiabatic rovibronic energy ($E_{n}^{\left(a\right)}$) computed with an $N_{\text{state}}$ model and the fully‐coupled 10‐state model presented in Section 3.1. The top left panel plots the energy level on the vertical axis vs. the discrepancy to the 10‐state computed energy, where the states in question reside in the potential region shown in the top right panel. The bottom panel illustrates the energy discrepancy with increasing number of electronic states for the lowest 4 vibrational states of $1^{1}\sum^{+}$.

To assess the validity of the two‐state approximation, we compare the lowest 25 rovibronic energy levels of the 10‐state system described in Section 3.1 by progressively reducing the number of electronic states included in the nuclear motion calculations. Figure 6 shows the absolute differences between the $n^{\text{th}}$ rovibronic energy level computed with an $N_{\text{state}}$ model and the full 10‐state adiabatic model with all DDR couplings, which we treat as the “true” reference for this system.

The most significant improvement occurs when moving from a single‐state model to the two‐state coupled system, reflected by an order‐of‐magnitude reduction in energy error, highlighting the critical role of nonadiabatic interactions. However, even for the lowest rovibronic states, the two‐state model never agrees with the 10‐state results to better than $10^{-2}$ cm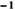, indicating the necessity of incorporating additional nonadiabatic interactions from higher excited electronic states. Even the 9‐state model does not achieve agreement with the 10‐state reference within $10^{-3}$ cm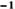, which is insufficient for high‐resolution spectroscopy.

Despite this argument being somewhat heuristic, it is evident that states separated by energies on the order of $10^{4}$ cm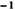 have a nonnegligible impact on the ground‐state energies. This can be attributed to the complex interactions between multiple different electronic states and results in a nontrivial correlation even among well‐separated states. This challenges the straightforward application of the Hellmann–Feynman theorem to truncate the number of adiabatic states treated in rovibronic applications and highlights the need for further investigation, which tools like duo now facilitate.

## 4 Conclusions

We demonstrate (for the first time) the numerical equivalence of the adiabatic and diabatic representations of nuclear motion for N‐state coupled diatomic systems using our rovibronic code duo. Adiabatically and diabatically computed rovibronic energies for the 3‐state ${\text{N}}_{2}$ [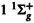, 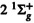, 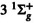] system, 4‐state CH [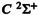, 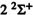, 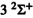, 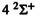] system, and an artificially generated 10‐state system were shown to be numerically exact. These results demonstrate that a strict diabatic representation, in which all DDR couplings vanish, can be achieved for systems with an arbitrary number of coupled states. Consequently, duo serves as a reliable benchmark for evaluating similar programs.

The importance of NACs, the DBOC, and the DC was shown numerically, where rovibronic energies are studied when omitting these terms from the molecular Hamiltonian. It is generally seen that the DCs for the N‐coupled state problem give rise to the most significant contribution for equivalency between the two representations. However, omission of any DDR coupling gives rise to significant changes in the computed rovibronic energies (and hence wavefunctions) up to $10^{2}$ cm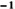 unsuitable for high‐resolution spectroscopy. duo then provides an efficient platform to test different aspects of diabatisations and different approximations for diatomic nuclear motion calculations. duo is an open‐access code, with an extended online manual and many examples.

We also demonstrate the problems of the 2‐state approximation, commonly argued for using Hellman–Feynman theorem, where the interaction to higher (technically infinite) excited electronic states is neglected. We studied the lowest rovibronic energy terms of the 10‐state system when removing progressively more states from the calculation and saw that, while the 2‐state approximation produces the most significant effect when going from a single‐ state model, all 10 states are required to reproduce a numerically complete spectroscopy of the ground state energies. Even the 9‐state model could not globally achieve equivalence with the 10‐state model energies to within $10^{-4}$ cm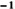 for the lowest rovibronic energy terms. Therefore, on top of the Hellman–Feynman theorem argument, quantitative investigation should be made before truncation of the number of adiabatic states in a diatomic calculation, and the corresponding error should be considered.

We provide all curves studied here on a grid of bond lengths in different ASCII files of the supplementary, which are also duo input files.

## Conflicts of Interest

The authors declare no conflicts of interest.

## Supporting information


**Data S1**: Supporting Information.

## Data Availability

The data that support the findings of this study are available in the Supporting Information of this article.

## References

[1] M. S. Schuurman and A. Stolow , “Dynamics at Conical Intersections,” Annual Review of Physical Chemistry 69 (2018): 427–450. 10.1146/annurev-physchem-052516-050721 29490199

[2] B. G. Levine and T. J. Martínez , “Isomerization Through Conical Intersections,” Annual Review of Physical Chemistry 58 (2007): 613–634. 10.1146/annurev.physchem.57.032905.104612 17291184

[3] J. Whitlow , Z. Jia , Y. Wang , C. Fang , J. Kim , and K. R. Brown , “Simulating Conical Intersections With Trapped Ions,” 2023 arXiv:2211.07319 [quant‐ph]. 10.1038/s41557-023-01303-0 37640856

[4] A. W. Jasper , C. Zhu , S. Nangia , and D. G. Truhlar , “Introductory Lecture: Nonadiabatic Effects in Chemical Dynamics,” Faraday Discussions 127 (2004): 1–22. 15471336 10.1039/b405601a

[5] S. Matsika and P. Krause , “Nonadiabatic Events and Conical Intersections,” Annual Review of Physical Chemistry 62 (2011): 621–643. 10.1146/annurev-physchem-032210-103450 21219147

[6] D. R. Yarkony , “Diabolical Conical Intersections,” Reviews of Modern Physics 68 (1996): 985–1013.

[7] Y. Shu , B. S. Fales , W.‐T. Peng , and B. G. Levine , “Understanding Nonradiative Recombination Through Defect‐Induced Conical Intersections,” Journal of Physical Chemistry Letters 8 (2017): 4091–4099. 28799771 10.1021/acs.jpclett.7b01707

[8] T. Karman , M. Besemer , A. van der Avoird , and G. C. Groenenboom , “Diabatic States, Nonadiabatic Coupling, and the Counterpoise Procedure for Weakly Interacting Open‐Shell Molecules,” Journal of Chemical Physics 148 (2018): 094105.

[9] T. Karman , A. van der Avoird , and G. C. Groenenboom , “Communication: Multiple‐Property‐Based Diabatization for Open‐Shell Van der Waals Molecules,” Journal of Chemical Physics 144 (2016): 121101. 27036418 10.1063/1.4944744

[10] Q. Ma , J. Kłos , M. H. Alexander , A. van der Avoird , and P. J. Dagdigian , “The Interaction of OH (X^2^II) With H2: Ab Initio Potential Energy Surfaces and Bound States,” Journal of Chemical Physics 141 (2014): 174309. 25381516 10.1063/1.4900478

[11] J. Kłos , Q. Ma , M. H. Alexander , and P. J. Dagdigian , “The Interaction of NO(X^2^II) With H_2_: Ab Initio Potential Energy Surfaces and Bound States,” Journal of Chemical Physics 146 (2017): 114301. 28330347 10.1063/1.4977992

[12] T. de Jongh , T. Karman , S. N. Vogels , et al., “Imaging Diffraction Oscillations for Inelastic Collisions of NO Radicals With He and D_2_ ,” Journal of Chemical Physics 147 (2017): 013918. 28688409 10.1063/1.4981023

[13] M. Baer , “Topological Effects in Molecular Systems: An Attempt Towards a Complete Theory,” Chemical Physics 259 (2000): 123–147.

[14] M. Baer and A. Alijah , “Quantized Non‐Adiabatic Coupling Terms to Ensure Diabatic Potentials,” Chemical Physics Letters 319 (2000): 489–493.

[15] M. Baer , “Introduction to the Theory of Electronic Non‐Adiabatic Coupling Terms in Molecular Systems,” Physics Reports 358 (2002): 75–142.

[16] M. Baer , Beyond Born‐Oppenheimer: Electronic Nonadiabatic Coupling Terms and Conical Intersections (John Wiley & Sons, 2006).

[17] F. T. Smith , “Diabatic and Adiabatic Representations for Atomic Collision Problems,” Physics Review 179 (1969): 111–123.

[18] D. R. Yarkony , C. Xie , X. Zhu , Y. Wang , C. L. Malbon , and H. Guo , “Diabatic and Adiabatic Representations: Electronic Structure Caveats,” Computational & Theoretical Chemistry 1152 (2019): 41–52.

[19] Z. Varga , K. A. Parker , and D. G. Truhlar , “Direct Diabatization Based on Nonadiabatic Couplings: The N/D Method,” Physical Chemistry Chemical Physics 20 (2018): 26643–26659. 30320314 10.1039/c8cp03410a

[20] W. Z. N. Mabrouk and H. Berriche , “Theoretical Study of the LiNa Molecule Beyond the Born‐Oppenheimer Approximation: Adiabatic and Diabatic Potential Energy Curves, Radial Coupling, Adiabatic Correction, Dipole Moments and Vibrational Levels,” Molecular Physics 118 (2020): e1605098.

[21] J. B. Delos , “Theory of Electronic Transitions in Slow Atomic Collisions,” Reviews of Modern Physics 53 (1981): 287–357.

[22] C. A. Mead and D. G. Truhlar , “Conditions for the Definition of a Strictly Diabatic Electronic Basis for Molecular Systems,” Journal of Chemical Physics 77 (1982): 6090–6098.

[23] A. W. Jasper , B. K. Kendrick , C. A. Mead , and D. G. Truhlar , “Non‐Born‐Oppenheimer Chemistry: Potential Surfaces, Couplings, and Dynamics,” in Modern Trends in Chemical Reaction Dynamics (World Scientific, 2004), 329–391.

[24] R. P. Brady , S. N. Yurchenko , G.‐S. Kim , W. Somogyi , and J. Tennyson , “An *Ab Initio* Study of the Rovibronic Spectrum of Sulphur Monoxide (SO): Diabatic vs. Adiabatic Representation,” Physical Chemistry Chemical Physics 24 (2022): 24076–24088. 36172791 10.1039/d2cp03051a PMC9623608

[25] J. Tennyson and S. N. Yurchenko , “ExoMol: Molecular Line Lists for Exoplanet and Other Atmospheres,” Monthly Notices of the Royal Astronomical Society 425 (2012): 21–33.

[26] J. Tennyson , S. N. Yurchenko , A. F. Al‐Refaie , et al., “The 2020 Release of the ExoMol Database: Molecular Line Lists for Exoplanet and Other Hot Atmospheres,” Journal of Quantitative Spectroscopy and Radiative Transfer 255 (2020): 107228.

[27] J. Tennyson , S. N. Yurchenko , J. Zhang , et al., “The 2024 Release of the ExoMol Database: Molecular Line Lists for Exoplanet and Other Hot Atmospheres,” Journal of Quantitative Spectroscopy and Radiative Transfer 326 (2024): 109083.

[28] Y. Shu , Z. Varga , A. G. Sampaio de Oliveira‐Filho , and D. G. Truhlar , “Permutationally Restrained Diabatization by Machine Intelligence,” Journal of Chemical Theory and Computation 17 (2021): 1106–1116. 33405927 10.1021/acs.jctc.0c01110

[29] R. P. Brady , C. Drury , S. N. Yurchenko , and J. Tennyson , “Numerical Equivalence of Diabatic and Adiabatic Representations in Diatomic Molecules,” Journal of Chemical Theory and Computation 20 (2024): 2127–2139. 38171539 10.1021/acs.jctc.3c01150 PMC10938500

[30] I. H. Zimmerman and T. F. George , “Numerical Comparison Between Electronically Adiabatic and Diabatic Representations for Collinear Atom‐Diatom Collisions,” Journal of Chemical Physics 63 (1975): 2109–2114.

[31] H.‐m. Shi , G.‐h. Guo , and Z.‐g. Sun , “Numerical Convergence of the Sinc Discrete Variable Representation for Solving Molecular Vibrational States With a Conical Intersection in Adiabatic Representation,” Chinese Journal of Chemical Physics 32 (2019): 333–342.

[32] L. Wolniewicz and K. Dressler , “The EF and GK States of Hydrogen: Adiabatic Calculation of Vibronic States in H_2_, HD, and D_2_ ,” Journal of Molecular Spectroscopy 67 (1977): 416.

[33] K. Dressler , R. Gallusser , P. Quadrelli , and L. Wolniewicz , “The EF and GK States of Hydrogen: Calculation of Nonadiabatic Coupling,” Journal of Molecular Spectroscopy 75 (1979): 205.

[34] K. Dressler and L. Wolniewicz , “Improved Adiabatic Corrections for the $B^{1}\sum_{u}^{+}$ $C^{1}\Pi_{u}$, and $D^{1}\Pi_{u}$ States of the Hydrogen Molecule and Vibrational Structures for H_2_, HD, and D_2_ ,” Journal of Chemical Physics 85 (1986): 2821.

[35] P. Quadrelli , K. Dressler , and L. Wolniewicz , “Nonadiabatic Coupling Between the EF+GK+H , I , and J States of the Hydrogen Molecule. Calculation of Rovibronic Structures in H2, HD, and D2,” Journal of Chemical Physics 92 (1990): 7461.

[36] L. Wolniewicz and K. Dressler , “Nonadiabatic Energy Corrections for the Vibrational Levels of the B and B' States of the H_2_ and D_2_ Molecules,” Journal of Chemical Physics 96 (1992): 6053.

[37] L. Wolniewicz and K. Dressler , “Adiabatic Potential Curves and Nonadiabatic Coupling Functions for the First Five Excited States of the Hydrogen Molecule,” Journal of Chemical Physics 100 (1994): 444.

[38] S. Yu and K. Dressler , “Calculation of Rovibronic Structures in the Lowest Nine Excited States of H_2_, D_2_, and T_2_ ,” Journal of Chemical Physics 101 (1994): 7692.

[39] K. Pachucki and J. Komasa , “Nonadiabatic Corrections to the Wave Function and Energy,” Journal of Chemical Physics 129 (2008): 034102. 18647011 10.1063/1.2952517

[40] K. Pachucki and J. Komasa , “Nonadiabatic Corrections to Rovibrational Levels of H_2_ ,” Journal of Chemical Physics 130 (2009): 164113. 19405567 10.1063/1.3114680

[41] M. V. Volkov , S. L. Yakovlev , E. A. Yarevsky , and N. Elander , “Adiabatic Versus Diabatic Approach to Multichannel Coulomb Scattering for Mutual Neutralisation Reaction $H^{+}+H^{-}\to H\left(1\right)+H\left(n\right)$ ,” Chemical Physics 462 (2015): 57.

[42] D. A. Little and J. Tennyson , “An R‐Matrix Study of Singlet and Triplet Continuum States of N_2_ ,” Journal of Physics B: Atomic, Molecular and Optical Physics 47 (2014): 105204.

[43] D. A. Little , K. Chakrabarti , I. F. Schneider , and J. Tennyson , “The Dissociative Recombination of $N_{2}^{+}$: An Ab Initio Study,” Physical Review A 90 (2014): 052705.

[44] N. Gelfand , K. Komarova , F. Remacle , and R. D. Levine , “On the Energy‐Specific Photodissociation Pathways of ^14^N_2_ and ^14^N^15^N Isotopomers to N Atoms of Different Reactivity: A Quantum Dynamical Perspective,” Astrophysical Journal 948 (2023): 58.

[45] R. P. Brady , “A Strict and Internally Consistent Diabatic Representation for Coupled *N*‐State Diatomics: A Hybrid Asymptotic‐Property‐Based Diabatization Method,” Journal of Chemical Physics 162 (2025): 174105. 40314262 10.1063/5.0260594

[46] T. Furtenbacher , S. T. Hegedus , J. Tennyson , and A. G. Császár , “Analysis of the Measured High‐Resolution Doublet Rovibronic Spectra of ^12^CH and ^16^OH,” Physical Chemistry Chemical Physics 24 (2022): 19287. 35929432 10.1039/d2cp02240k PMC9382695

[47] D. L. Lambert , “The Abundances of the Elements in the Solar Photosphere ‐ VIII. Revised Abundances of Carbon, Nitrogen and Oxygen,” Monthly Notices of the Royal Astronomical Society 182 (1978): 249–272.

[48] F. Mélen , N. Grevesse , A. Sauval , et al., “A New Analysis of the Vibration‐Rotation Spectrum of CH From Solar Spectra,” Journal of Molecular Spectroscopy 134 (1989): 305–313.

[49] N. Grevesse , D. Lambert , A. Sauval , E. van Dishoeck , C. Farmer , and R. Norton , “Vibration‐Rotation Bands of CH in the Solar Infrared Spectrum and the Solar Carbon Abundance,” Astronomy and Astrophysics 242 (1991): 488.

[50] S. T. Ridgway , D. F. Carbon , D. N. B. Hall , and J. Jewell , “An Atlas of Late‐Type Stellar Spectra, 2400‐2778 Inverse Centimeters,” Astrophysical Journal Supplement Series 54 (1984): 177.

[51] D. L. Lambert , B. Gustafsson , K. Eriksson , and K. H. Hinkle , “The Chemical Composition of Carbon Stars. I. Carbon, Nitrogen, and Oxygen in 30 Cool Carbon Stars in the Galactic Disk,” Astrophysical Journal. Supplement Series 62 (1986): 373.

[52] T. Masseron , B. Plez , S. Van Eck , et al., “CH in Stellar Atmospheres: An Extensive Linelist,” Astronomy and Astrophysics 571 (2014): A47.

[53] M. Womack , B. L. Lutz , and R. M. Wagner , “Pre‐ and Postperihelion Abundances of Gas and Dust in Comet Halley,” Astrophysical Journal 433 (1994): 886.

[54] P. Swings and L. Rosenfeld , “Considerations Regarding Interstellar Molecules,” Astrophysical Journal 86 (1937): 483.

[55] M. Jura and D. M. Meyer , “An Optical Measurement of the Population Inversion of the Ground State Lambda Doublet of Interstellar CH,” Astrophysical Journal 294 (1985): 238.

[56] W. B. Somerville and I. A. Crawford , “Observations of Molecules in Diffuse Interstellar Clouds,” Journal of the Chemical Society, Faraday Transactions 89 (1993): 2261.

[57] D. Lien , “A Reanalysis of the Interstellar CH Abundance,” Astrophysical Journal 284 (1984): 578.

[58] G. J. Stacey , J. B. Lugten , and R. Genzel , “Detection of Interstellar CH in the Far‐Infrared,” Astrophysical Journal 313 (1987): 859.

[59] D. F. Strobel , “Chemistry and Evolution of Titan's Atmosphere,” Planetary and Space Science 12 (1982): 244.

[60] R. Meier , J. A. Samson , Y. Chung , E.‐M. Lee , and Z.‐X. He , “Production of N^+^* From N_2_ + *hν*: Effective EUV Emission Yields From Laboratory and Dayglow Data,” Planetary and Space Science 39 (1991): 1197.

[61] R. P. Wayne , Chemistry of Atmospheres, 3rd ed. (Oxford University Press, 2000).

[62] D. C. Knauth , B.‐G. Andersson , S. R. McCandliss , and H. W. Moos , “The Interstellar N_2_ Abundance Towards HD 124314 From Far‐Ultraviolet Observations,” Nature 429 (2004): 636. 15190346 10.1038/nature02614

[63] F. R. Gilmore , “Potential Energy Curves for N_2_, NO, O_2_ and Corresponding Ions,” Journal of Quantitative Spectroscopy and Radiation Transfer 5 (1965): 369.

[64] J. Jiang , H.‐Z. Ye , K. Nauta , T. Van Voorhis , T. W. Schmidt , and R. W. Field , “Diabatic Valence‐Hole States in the C_2_ Molecule: “Putting Humpty Dumpty Together Again”,” Journal of Physical Chemistry. A 126 (2022): 3090. 35544770 10.1021/acs.jpca.2c00495

[65] J. Jiang , “Diabatic Valence‐Hole Concept,” Journal of Physical Chemistry. A 128 (2024): 3253–3265. 38647413 10.1021/acs.jpca.4c00289

[66] J. Römelt , “A Hermitean Reformulation of the Born‐Oppenheimer Nonadiabatic Coupling Terms for Diatomic Molecules,” International Journal of Quantum Chemistry 24 (1983): 627–631.

[67] B. D. Esry and H. R. Sadeghpour , “Split Diabatic Representation,” Physical Review A 68 (2003): 042706.

[68] G. W. Richings and G. A. Worth , “A Practical Diabatisation Scheme for Use With the Direct‐Dynamics Variational Multi‐Configuration Gaussian Method,” Journal of Physical Chemistry. A 119 (2015): 12457–12470. 26422169 10.1021/acs.jpca.5b07921

[69] S. N. Yurchenko , L. Lodi , J. Tennyson , and A. V. Stolyarov , “Duo: A General Program for Calculating Spectra of Diatomic Molecules,” Computer Physics Communications 202 (2016): 262–275.

[70] R. Guardiola and J. Ros , “On the Numerical Integration of the Schrödinger Equation in the Finite‐Difference Schemes,” Journal of Computational Physics 45 (1982): 374–389.

[71] J. R. Lund and B. V. Riley , “A Sine‐Collocation Method for the Computation of the Eigenvalues of the Radial Schrödinger Equation,” IMA Journal of Numerical Analysis 4 (1984): 83–98.

[72] J. Lo and B. D. Shizgal , “Spectral Convergence of the Quadrature Discretization Method in the Solution of the Schrödinger and Fokker‐Planck Equations: Comparison With Sinc Methods,” Journal of Chemical Physics 125 (2006): 194108. 17129090 10.1063/1.2378622

[73] W. H. Press , S. A. Teukolsky , W. T. Vetterling , and B. P. Flannery , Numerical Recipes–The Art of Scientific Computing, 3rd ed. (Cambridge University Press, 2007).

[74] H. Hellmann , “Zur rolle der kinetischen elektronenenergie for die zwischenatomaren kröfte,” European Physical Journal A 85 (1933): 180.

[75] R. P. Feynman , “Forces in Molecules,” Physics Review 56 (1939): 340–343.

[76] S. Fatehi , E. Alguire , Y. Shao , and J. E. Subotnik , “Analytic Derivative Couplings Between Configuration‐Interaction‐Singles States With Built‐In Electron‐Translation Factors for Translational Invariance,” Journal of Chemical Physics 135 (2011): 234105. 22191862 10.1063/1.3665031

[77] R. Gherib , L. Ye , I. G. Ryabinkin , and A. F. Izmaylov , “On the Inclusion of the Diagonal Born‐Oppenheimer Correction in Surface Hopping Methods,” Journal of Chemical Physics 144 (2016): 154103. 27389205 10.1063/1.4945817

[78] P. Habitz and C. Votava , “The Hellmann–Feynman Theorem for Approximate Wave Functions and Its Application to Nonadiabatic Coupling Matrix Elements With the Aid of a Coupled Hartree–Fock Method,” Journal of Chemical Physics 72 (1980): 5532–5539.

[79] A. V. Stolyarov and M. S. Child , “Analog of the Hellmann‐Feynman Theorem in Multichannel Quantum‐Defect Theory,” Physical Review A 63 (2001): 052510, 10.1103/PhysRevA.63.052510.

[80] V. I. Pupyshev , E. A. Pazyuk , A. V. Stolyarov , M. Tamanis , and R. Ferber , “Analogue of Oscillation Theorem for Nonadiabatic Diatomic States: Application to the *A* ^1^∑^+^ and *b* ^3^Π States of KCs,” Physical Chemistry Chemical Physics 12 (2010): 4809. 20428562 10.1039/b918384a

[81] V. Pupyshev , E. Pazyuk , A. Stolyarov , M. Tamanis , and R. Ferber , “On Oscillation Theorem for Two‐Component Schrödinger Equation, arXiv,” (2009), 10.48550/arXiv.0907.1380.

[82] N. Gelfand , F. Remacle , and R. D. Levine , “Recombination of N Atoms in a Manifold of Electronic States Simulated by Time‐Reversed Nonadiabatic Photodissociation Dynamics of N_2_ ,” Journal of Physical Chemistry Letters 14 (2023): 4625. 37166125 10.1021/acs.jpclett.3c00666 PMC10201567

[83] J. Z. Zhi Qin and L. Liu , “Radiative Transition Probabilities Between Low‐Lying Electronic States of N_2_ ,” Molecular Physics 117 (2019): 2418.

[84] E. F. van Dishoeck , “Photodissociation Processes in the CH Molecule,” Journal of Chemical Physics 86 (1987): 196–214.

[85] A. Kalemos , A. Mavridis , and A. Metropoulos , “An Accurate Description of the Ground and Excited States of CH,” Journal of Chemical Physics 111 (1999): 9536–9548.

[86] S. N. Yurchenko , E. Nogué , A. A. A. Azzam , and J. Tennyson , “ExoMol Line Lists ‐ XLVII. Rovibronic Spectrum of Aluminium Monochloride (AlCl),” Monthly Notices of the Royal Astronomical Society 520 (2022): 5183.

[87] M. Pezzella , J. Tennyson , and S. N. Yurchenko , “ExoMol Photodissociation Cross‐Sections ‐ I. HCl and HF,” Monthly Notices of the Royal Astronomical Society 514 (2022): 4413.

[88] T. Uhlikova , S. N. Yurchenko , A. N. Perri , J. Tennyson , and G.‐S. Kim , “Photodissociation Cross Sections and Quasi‐Continuum Properties of the NH Radical,” Journal of Chemical Physics 162 (2025): 144108. 40202144 10.1063/5.0262447

[89] I. Lengsfield , H. Byron , and D. R. Yarkony , “On the Evaluation of Nonadiabatic Coupling Matrix Elements for MCSCF/CI Wave Functions Using Analytic Derivative Methods. III. Second Derivative Terms,” Journal of Chemical Physics 84 (1986): 348.

[90] P. Saxe and D. R. Yarkony , “On the Evaluation of Nonadiabatic Coupling Matrix Elements for MCSCF/CI Wave Functions. IV. Second Derivative Terms Using Analytic Gradient Methods,” Journal of Physical Chemistry 86 (1987): 321–328.

